# Prevalence of Right Ventricle Strain Changes following Anthracycline Therapy

**DOI:** 10.3390/life12020291

**Published:** 2022-02-15

**Authors:** Michal Laufer-Perl, Moran Perelman-Gvili, Svetlana Sirota Dorfman, Guy Baruch, Ehud Rothschild, Gil Beer, Yaron Arbel, Joshua H. Arnold, Zach Rozenbaum, Shmuel Banai, Yan Topilsky, Livia Kapusta

**Affiliations:** 1 Department of Cardiology, Tel Aviv Sourasky Medical Center, 6 Weizman Street, Tel Aviv 6423906, Israel; svetasirota13@gmail.com (S.S.D.); gn.baruch@gmail.com (G.B.); ehud.rothschild@gmail.com (E.R.); yarona@tlvmc.gov.il (Y.A.); zachr@tlvmc.gov.il (Z.R.); shmuelb@tlvmc.gov.il (S.B.); yant@tlvmc.gov.il (Y.T.); 2 Sackler School of Medicine, Tel Aviv University, Ramat Aviv, P.O. Box 39040, Tel Aviv 6997801, Israel; gilbe@tlvmc.gov.il (G.B.); joshuaharnold1@gmail.com (J.H.A.); livia.kapusta@gmail.com (L.K.); 3 Internal Medicine T, Tel Aviv Sourasky Medical Center, 6 Weizman Street, Tel Aviv 6423906, Israel; 4 Faculty of Health Sciences, Ben-Gurion University of the Negev, Beer Sheva 8410501, Israel; 5 Pediatric Cardiology Unit, Tel Aviv Sourasky Medical Center, 6 Weizman Street, Tel Aviv 6423906, Israel; 6 Department of Medicine, University of Illinois at Chicago, 1853 W. Polk (MC 785), Chicago, IL 60612-7332, USA; 7 Department of Pediatric Cardiology, Amalia Children’s Hospital, Radboud University Medical Centre, Greet Grooteplein Zuid 32, 6525 Nijmegen, The Netherlands

**Keywords:** anthracycline, cardio-oncology, cardiotoxicity, strain, right ventricle

## Abstract

Background: Anthracycline (ANT) is the most recognized therapy known to cause cardiotoxicity, mainly left ventricle (LV) dysfunction. Global Longitudinal Strain (GLS) is the optimal tool for assessment of subclinical LV dysfunction. Right ventricle (RV) function has been recognized as an independent factor for cardiac outcomes; however, data evaluating RV GLS is limited. We aimed to evaluate the change in RV GLS following ANT therapy. Methods: The study cohort is part of the Israel Cardio-Oncology Registry (ICOR). All patients performed echocardiography before (T1) and at the end (T3) of ANT therapy. A significant reduction was defined as a relative reduction of ≥10% in RV GLS values. Results: The study included 40 female patients with breast cancer treated with ANT. During follow-up, both RV GLS and free wall longitudinal strain systolic peak (RV FWLS PK) decreased significantly (*p* < 0.001 and *p* = 0.002). Altogether, 30 (75%) and 23 (58%) patients showed RV GLS and RV FWLS PK ≥ 10% relative reduction. At T3, LV ejection fraction and LV GLS were within normal range. Conclusions: RV GLS and RV FWLS PK reduction following ANT exposure is extremely frequent, comparing to LV GLS reduction.

## 1. Introduction

Advancements in early diagnosis and therapy for breast cancer have significantly improved overall patient morbidity and mortality, however, at the cost of short and long-term side effects [1,2]. Cardiotoxicity, a significant complication developing from exposure to cancer therapeutics, has also been shown to lead to marked rates of morbidity and mortality, occasionally, even more so than cancer itself [3,4]. Anthracycline (ANT), specifically doxorubicin, is still the standard of care therapy for patients diagnosed with breast cancer [4]. ANT’s anti-tumor effect can lead to damage and death of cardiac myocytes, causing type I toxic cardiomyopathy, which is characterized by dose-dependent irreversible cell death [5,6]. Early detection of ANT-related cardiomyopathy may allow for prevention of the development of significant left ventricle (LV) dysfunction and heart failure by allowing clinicians to alter the cancer therapy regimen or to introduce cardio-protective treatments [7]. Thus, the need for early detection of cardiac damage is paramount [6,7,8]. Echocardiography is the most common tool utilized for the diagnosis and tracking of cardiac dysfunction in cancer patients, with changes in LV ejection fraction (LVEF) used as the main reference point [5]. However, as LVEF reduction will only be evident in the presence of significant myocardial damage, the search for a more sensitive tool for subclinical changes is ongoing [9]. Two-dimensional (2D) speckle tracking echocardiography (2D-STE), specifically Global Longitudinal Strain (GLS), is emerging as such a tool for the assessment of subclinical and overall cardiac function. GLS is the optimal parameter for early detection of LV dysfunction and has been found to precede and predict LVEF changes by echocardiography and Magnetic Resonance Imaging (MRI) [10,11,12,13]. While the LV is currently the focus in cardiac function evaluation, right ventricle (RV) function has also been recognized as an independent prognostic factor in many cardiovascular (CV) diseases [14]. A number of studies have shown that the RV is similarly negatively affected by ANT exposure [15,16] with a decrease in overall RV function. To date, there are a number of different parameters for the evaluation of RV function [17,18], such as tricuspid annular plane systolic excursion (TAPSE) and tricuspid S’ velocity. However, none of these existing parameters are considered optimal for the evaluation of RV function, and more accurate methods are needed. Recently, RV 2D-STE has been used as an objective and accurate tool in the evaluation of RV function, and is a valuable asset in recognizing early subclinical changes in the myocardium [17,19]. There are limited studies evaluating the use of RV 2D-STE for routine follow-up in cancer patient populations. We aim, in our pilot study, to evaluate the changes in RV 2D-STE following ANT therapy in patients with breast cancer.

## 2. Materials and Methods

### 2.1. Study Population

The cohort is part of the Israel Cardio-Oncology Registry (ICOR) [20,21]—a prospective registry enrolling cancer patients visiting the cardio-oncology clinic at the Tel Aviv Sourasky Medical Center. Patients included in the registry signed an informed consent form at their first visit. The registry was approved by the local ethics Tel Aviv Sourasky committee (Identifier: 0228-16-TLV). Inclusion criteria were female breast cancer patients treated with doxorubicin who performed two echocardiography exams; at baseline (T1) and after the completion of doxorubicin therapy (T3). The majority of patients also performed echocardiography exam following receipt of a cumulative dose of 180 mg/m^2^ of doxorubicin (T2). Exclusion criteria included age below 18, non-sinus rhythm, baseline LVEF < 53%, a history of cardiac disease, past ANT exposure, dexrazoxane therapy, doxorubicin dose < 180 mg/m^2^, or poor RV image quality for strain analysis.

A significant reduction in RV 2D-STE was defined as a relative reduction of ≥10% in either RV GLS or RV FWLS PK, as considered clinically significant among the definitions of LV GLS reduction [22].

### 2.2. Study Protocol

**Figure 1:**
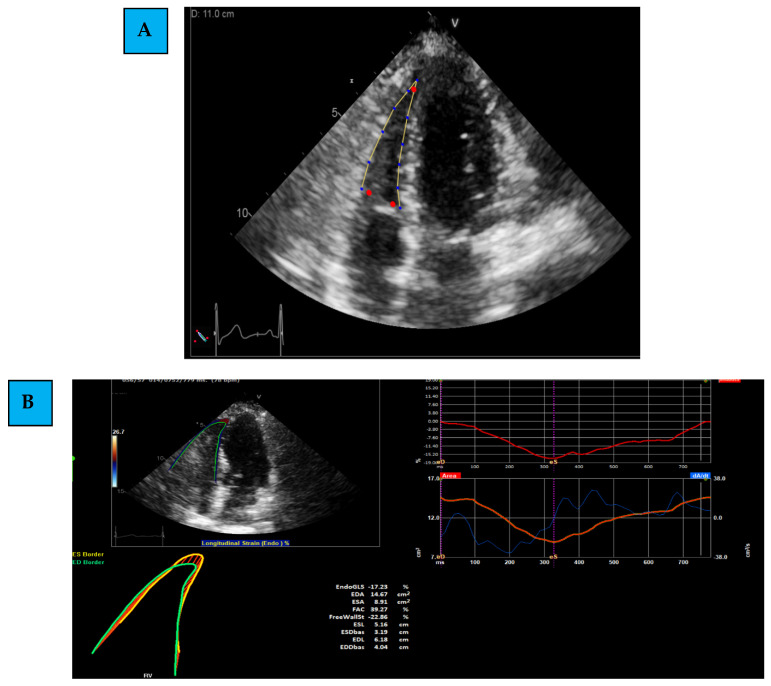

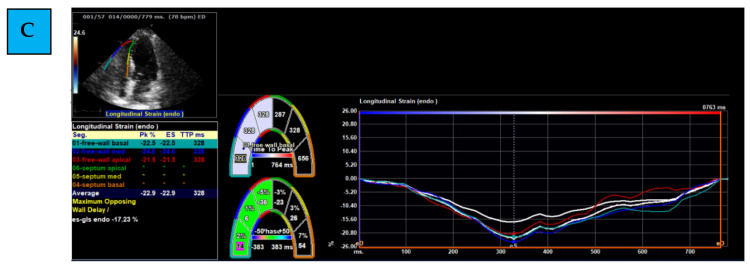
The assessment of right ventricle Global Longitudinal Strain. (**A**) Determining of the region of interest; (**B**) evaluating the Global Longitudinal Strain of the right ventricle; (**C**) evaluating the free wall by excluding the septal wall and including the RV free wall (basal, mid, and apical) segments solely.

Medical history and treatment were collected from the electronic medical charts. Echocardiographic examinations were performed using the same protocol, personnel, and equipment (General Electric (GE) system, Haifa, Israel, model Vivid S70). LV echocardiographic measures included LV diameters, and LVEF [23]. TAPSE was measured as the distance of systolic excursion of the RV annular segment along its longitudinal plane, using M-mode from the apical 4C window [18]. Images were acquired using a high frame rate (>50 frames/s) [24]. LV GLS was measured using EchoPac STE software. LV boundaries were assessed at the end-systolic frame, which was first measured by automatic tracking and was later followed by performing manual corrections and optimization of images. We then performed offline measurements of RV 2D-STE by TOMTEC software, using the 2D PCA (cardiac performance analysis) application. The assessment included the following steps, as accepted by the most updated literature [25,26]: (Figure 1A–C)

Choosing of the most optimal, best quality, apical 4C RV-focused view image.

(1) Determining the region of interest (ROI): using the apical 4C RV-focused view, the margins of the RV are marked. This is first determined through automatic identification (by the software), and later, manual corrections and modifications are performed.
(2) Evaluating Global and Regional Longitudinal Strain: RV GLS is the average of 6 segments (3 of the free wall and 3 of the septum) (Figure 1B). Each wall of the RV is divided into 3 equal parts from the base to the apex. RV FWLS PK is, for example, the average measured strain of the basal, mid, and apical segments of the RV FWLS PK solely (Figure 1C).
(3) Determining the timing of measurement: strain can be measured during several phases of the cardiac cycle: End Systolic (ES) strain (defined by pulmonary valve closure), Peak (PK) systolic strain (maximal ventricular contraction), or Peak strain (the highest value throughout the entire heart cycle). To date, PK systolic value is the recommended measurement to use [22].

### 2.3. Statistical Analysis

The sample size was calculated in reference to previous data measured in our cohort, showing that ANT therapy led to a clinically significant reduction in LV GLS values among 30% of the patients. Accordingly, we expected similar changes in RV GLS values to occur in 30% of our patients. When calculating the sample size for this trial with alpha-0.05 and 80% power, we found that we needed to include 19 patients. To avoid under-powering in our study, we chose to enroll 40 patients. The IBM SPSS Statistics 25.0 software was used. Continuous normally distributed variables are described as means ± standard deviation and continuous not normally distributed variables are described as medians (Q1, Q3). Categorical variables are described as percentages. Parametric data from two different groups were compared using the Mann–Whitney test and *t*-test, for two related groups using paired *t*-test, and repeated measures ANOVA for three groups. Nonparametric data from two related groups were compared using Wilcoxon matched-pair test and Friedman’s 2-way ANOVA for three related groups. Categorical variables were compared using a Chi square test. Pearson and Spearman correlation analysis was used for continuous parametric and nonparametric data, respectively. *p*-values < 0.05 were considered statistically significant.

## 3. Results

Eighty-five patients were evaluated for this study between September 2016 to 2019, of which 40 were included in our cohort. A total of 45 patients were excluded: 29 for poor echocardiography image quality of the RV, 13 for incompatible timing of echocardiographic exam, and 3 for not completing the ANT protocol therapy.

The mean age was 50 (±13) years, and cardiovascular risk factors were relatively uncommon, ranging from 5% to 20%. A total of 10 (25%) patients were treated with cardio-protective drugs, as specified in Table 1.

The mean cumulative dose of doxorubicin was 238.5 (±9.4) mg/m^2^. The mean time elapsed from last doxorubicin treatment to T3 was 96 ± 22 days. At T3, 10 (25%) patients were treated with trastuzumab (humanized anti-Human Epidermal Growth Factor Receptor 2 (HER2) monoclonal), 9 (22.5%) with pertuzumab (a humanized anti-HER2 monoclonal) and 18 (45%) patients had undergone chest radiations as shown in Table 1.

All patients had normal baseline LVEF, LV GLS, and TAPSE (Table 1). RV baseline 2D-STE parameters included mean RV GLS −26.8 (±4.7)%, mean RV FWLS PK −28.9 (±5.1)%, and median RV GLS septum PK −23.6 (20.2, 28.0)%.

Two observers (M.P.G. and L.K.) measured the RV GLS in order to assess interobserver variability. Intraobserver variability was assessed by M.P.G. at an interval of 2–3 weeks. Evaluating 10 patients, we found a high level of agreement with an interobserver correlation coefficient of 0.969 and an intraobserver correlation coefficient of 0.847.

**Figure 2:**
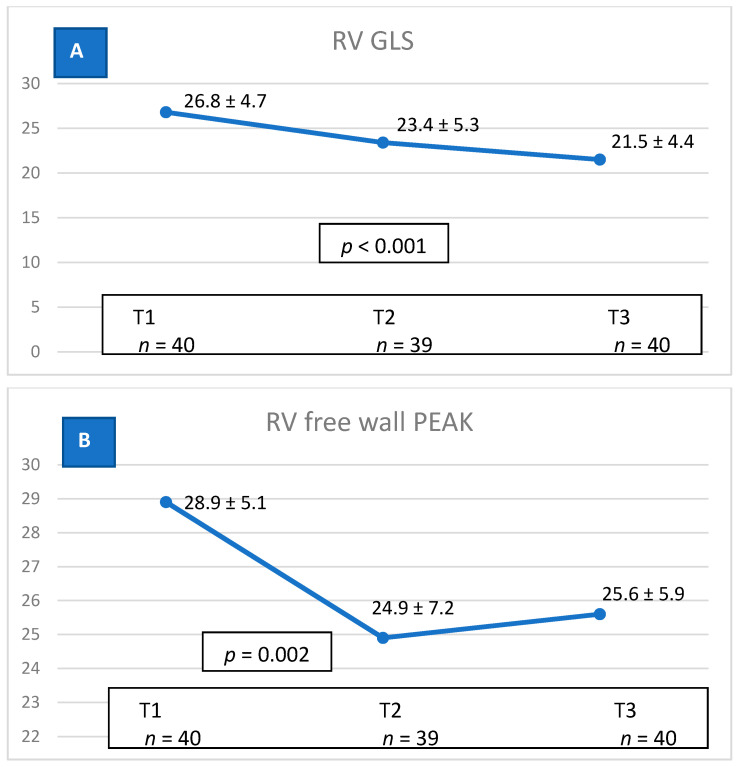

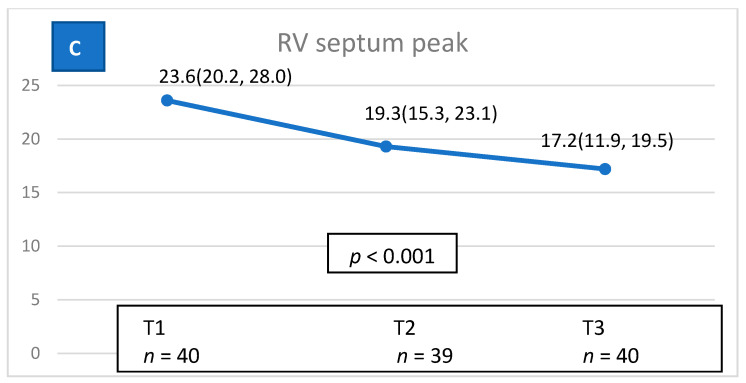
Right ventricle strain reduction during ANT therapy. (**A**) Reduction in RV GLS values from T1 to T3; (**B**) reduction in RV FWLS PK values from T1 to T3; (**C**) reduction in RV GLS septum PK values form T1 to T3. ANT = Anthracycline, RV = Right Ventricle, GLS = Global Longitudinal Strain, FWLS PK = Free Wall Peak Systolic, PK = Peak Systolic, T1 = baseline before ANT therapy, T2 = during ANT therapy, T3 = end of ANT therapy.

The mean RV GLS reduced significantly from −26.8 (±4.7)% at T1 to −21.5 (±4.4)% at T3 (*p* < 0.001). Both the mean RV FWLS PK and median RV GLS septum PK reduced significantly from −28.9 (±5.1)% at T1 to −25.6 (±5.9)% at T3 (*p* = 0.002) and from −23.6 (20.2, 28.0)% at T1 to −17.2 (11.9, 19.5)% at T3 (*p* < 0.001), respectively (Figure 2A–C). At T3, 30 (**75**%) and 23 (58%) patients showed a significant ≥ 10% relative reduction in RV GLS and RV FWLS PK, respectively. RV GLS and RV FWLS PK relative reduction of ≥ 15% was seen in 28 (70%) and 20 (50%) of the patients, respectively (Table 2). 

At T3, LVEF and LV GLS were within the normal range (Table 2). While LV GLS 10% and 15% relative reduction were observed in 14 (35%) and 8 (20%) patients, LVEF reduction of 10% and above developed in only 2 (5%) patients, and none of the patients presented with heart failure. No correlation between RV GLS relative reduction and LV GLS relative reduction was observed. No significant differences were observed regarding echocardiographic parameters, including RV GLS and RV FWLS PK, at T3 between patients treated with or without trastuzumab therapy (*p* = 0.850 and *p* = 0.901, respectively).

## 4. Discussion

In this pilot study, we demonstrated a significant and frequent reduction in both RV GLS and RV FWLS PK in breast cancer patients exposed to ANT therapy. While RV GLS reduction was extremely frequent, LV function remained within normal values, suggesting that the RV may be more vulnerable to ANT therapy. Future studies are needed to evaluate whether early RV dysfunction can be used as a marker for LV dysfunction development in cancer patients.

Overall RV function has been recognized as an independent prognostic factor in a number of CV diseases [14] and has recently been shown to be affected by ANT exposure [15,16]. RV function is routinely assessed by measuring sub-optimal estimations, mainly TAPSE and tricuspid S’ velocity [17,18]. In contrast to standard echocardiography methods, RV 2D-STE is able to evaluate the intrinsic function of the myocardium and can distinguish passive from active motion of individual myocardial fibers [19,27]. Moreover, 2D-STE is less angle-dependent, less susceptible to artifact, and easily performed. RV 2D-STE can also be assessed from a single apical 4C view, which is routinely performed. Planek et al. [28] showed that in patients with lymphoma, doxorubicin therapy was associated with a reduction in RV strain values, despite no change in LV function. Calle et al. [29] also showed that early changes in GLS acts as a predictor for the development of cardiotoxicity in patients treated with anthracycline and trastuzumab. Similar results were shown by Zhao et al. [30], in which the decrease in RVEF preceded LVEF changes. One of the reasons suggested by these authors relates to the fact that the RV wall is thinner, having less reserve for compensation compared to the LV, and is thus more susceptible to toxicity, leading to earlier manifestations. This finding is consistent with other imaging modalities, as evidenced by a study from Ylanen et al. [31] evaluating the late effects of ANT in childhood cancer survivors, using cardiac MRI. This study showed a 27% prevalence of abnormal RV function and only an 18% prevalence of abnormal LV function among these patients. In concordance with those results, our study found that a reduction in both RV GLS and RV FWLS PK were extremely frequent (75% and 58%, respectively), and reduced significantly during ANT therapy. Furthermore, this reduction occurred early, within a mean of 138 days from T1. When looking at the change in RV GLS values, both a 10% and 15% relative reduction were frequent, ranging from 75% and 70% for RV GLS and 58% and 50% for RV FWLS PK. When compared to the LV GLS 10% and 15% relative reduction observed among 35% and 20% of patients, we are able to deduce that the RV is more susceptible to myocyte damage. No correlation was observed between RV GLS and LV GLS relative reduction, which may also suggest that RV GLS changes precede change in LV GLS. This further emphasizes the importance of screening for early subclinical RV dysfunction in cancer patients, even in the presence of normal LV GLS values.

The development of RV dysfunction has recently been the focus of other cancer therapy regimens. For example, Calleja et al. [32] demonstrated the development of RV dysfunction in breast cancer patients treated with trastuzumab. However, these patients mostly had concurrent LV dysfunction, in contrast to our pilot study which was developed to observe the use of RV GLS as an early subclinical marker prior to the development of LV dysfunction. Keramida et al. [33] showed a reduction in RV GLS and RV FWLS PK six months following the initiation of trastuzumab therapy, with a relative reduction of −14.8% considered a predictive factor for cardiotoxicity development. In contrast, we did not observe any significant differences in RV 2D-STE values between patients treated with or without trastuzumab. This might be explained by the fact that our cohort focused on ANT therapy with shorter follow-up evaluation, a period of time in which patients typically received only 1–2 treatments of trastuzumab. Similarly, Keramida et al. [33] showed no change in RV 2D-STE at 3-months follow-up.

Monitillo et al. [34] showed that RV dysfunction is a marker of poor prognosis, regardless of LV function. Bosch et al. [35] also showed that RV systolic dysfunction was independently associated with lower LVEF. Due to the short follow-up time, we did not analyze the association between RV 2D-STE reduction and future LV dysfunction. Moreover, due to the small size of our cohort, we could not perform multivariable adjustments and take into account confounders such as blood pressure and other medical treatment.

Our study has several limitations. First, it is a single center study, and as such may have been subject to bias. However, the prospective nature, homogenous population, and the fact that echocardiography assessment was done by the same vendor, technician, and interpreting physician is an advantage. Second, the relatively small cohort reduces the statistical power and larger trials are needed. Third, the short period of time for follow up did not allow us to evaluate whether the reduction in RV 2D-STE values is permanent, or whether a recovery will occur. Furthermore, due to the low events rate of morbidity and mortality, we were unable to evaluate the clinical association between RV 2D-STE reduction and CV outcomes.

## 5. Conclusions

Our pilot study examines an understudied echocardiography technique for the early detection and diagnosis of sub-clinical cardiac damage in cancer patients. A significant reduction in RV GLS and RV FWLS PK is extremely frequent among patients diagnosed with breast cancer and exposed to ANT therapy. Furthermore, we showed that RV GLS reduction was more frequent than LV GLS reduction. Larger studies with longer follow-up are needed to evaluate the impact of RV GLS measurement in routine follow-up and the need for initiating cardio-protective therapies among patients treated with ANT.

## Figures and Tables

**Table 1:** Baseline characteristics.

Variables	All Patients (40)
Age (years) (mean, SD)	50 (±13)
Hypertension (*n*, %)	8 (20)
Ischemic heart disease (*n*, %)	0 (0)
Diabetes mellitus (*n*, %)	2 (5)
Chronic heart failure (*n*, %)	0 (0)
Chronic kidney disease (*n*, %)	0 (0)
Hyperlipidemia (*n*, %)	5 (12.5)
Smoker (*n*, %)	
No	25 (62.5)
Yes	9 (22.5)
Past Smoker	6 (15)
ACEi (*n*, %)	2 (5)
ARB (*n*, %)	4 (10)
BB (*n*, %)	4 (10)
ACEi/ARB/BB (yes)	6 (15.0)
Statins (*n*, %)	5 (12.5)
Trastuzumab (*n*, %)	10 (25)
Pertuzumab (*n*, %)	9 (22.5)
Chest Radiation (*n*, %)	18 (45)
Ejection fraction (%) (mean, SD)	60 (±0)
Left Ventricle Global Longitudinal strain (%) (mean, SD)	−21.5 (±2)
RV GLS (mean, SD)	26.8 (±4.7)
RV FWGLS PK (mean, SD)	28.9 (±5.1)
RV GLS septum PK (Median (Q1, Q3))	23.6 (20.2, 28.0)
TAPSE (mean, SD)	25 ± 3
SPAP (15/40) (mean, SD)	26 ± 6

**Table 2:** Study outcomes at T3.

Variables	Number(%)
RV GLS 10% relative reduction (*n*, %)	30 (75)
RV FWLS PK 10% relative reduction (*n*, %)	23 (58)
RV GLS septum PK 10% relative reduction (*n*, %)	31 (78)
RV GLS 15% relative reduction (*n*, %)	28 (70)
RV FWLS PK 15% relative reduction (*n*, %)	20 (50)
RV GLS septum PK 15% relative reduction (*n*, %)	29 (73)
LV GLS (%) (mean, SD)	19.7 (±1.8)
EF (%) (mean, SD)	59 (±2)
LV GLS 10% relative reduction (*n*, %)	14 (35)
LV GLS 15% relative reduction (*n*, %)	8 (20)
CTRCD (*n*, %)	2 (5)

GLS = Right Ventricle Global Longitudinal Strain, RCFWLS = Right Ventricle Free Wall Longitudinal Strain, PK = Peak systolic, T3 = echocardiography after the completion of doxorubicin therapy, LVGLS = Left Ventricle Global Longitudinal Strain, LVEF = Left Ventricle Ejection Fraction, CTRCD = Cancer Therapeutics-Related Cardiac Dysfunction.

## Data Availability

Not applicable.
